# The Prevalence and Impact of Coinfection and Superinfection on the Severity and Outcome of COVID-19 Infection: An Updated Literature Review

**DOI:** 10.3390/pathogens11040445

**Published:** 2022-04-07

**Authors:** Samya A. Omoush, Jihad A. M. Alzyoud

**Affiliations:** 1 Department of Medical Laboratory Sciences, Faculty of Applied Medical Sciences, The Hashemite University, P.O. Box 330127, Zarqa 13133, Jordan; samyah@hu.edu.jo; 2 Faculty of Medicine, The Hashemite University, P.O. Box 330127, Zarqa 13133, Jordan

**Keywords:** SARS-CoV-2, COVID-19, coinfection, mortality

## Abstract

Patients with viral illness are at higher risk of secondary infections—whether bacterial, viral, or parasitic—that usually lead to a worse prognosis. In the setting of Corona Virus Disease 2019 (COVID-19), the Severe Acute Respiratory Syndrome Coronavirus-type 2 (SARS-CoV-2) infection may be preceded by a prior microbial infection or has a concurrent or superinfection. Previous reports documented a significantly higher risk of microbial coinfection in SARS-CoV-2-positive patients. Initial results from the United States (U.S.) and Europe found a significantly higher risk of mortality and severe illness among hospitalized patients with SARS-CoV-2 and bacterial coinfection. However, later studies found contradictory results concerning the impact of coinfection on the outcomes of COVID-19. Thus, we conducted the present literature review to provide updated evidence regarding the prevalence of coinfection and superinfection amongst patients with SARS-CoV-2, possible mechanisms underlying the higher risk of coinfection and superinfection in SARS-CoV-2 patients, and the impact of coinfection and superinfection on the outcomes of patients with COVID-19.

## 1. Introduction

In December 2019, a third coronavirus outbreak was reported in China, known as "the Corona Virus Disease 2019 (COVID-19)", which causes severe respiratory illness and has rapidly evolved to spread [1,2] as a global pandemic that overwhelmed the capacity of healthcare systems in many countries, leading to substantial healthcare and socioeconomic implications [1]. The high replication rate of the Severe Acute Respiratory Syndrome Coronavirus-type 2 (SARS-CoV-2), the causative agent of COVID-19, and the high proportion of asymptomatic patients has led to constant growth in the active cases rate and increased healthcare services demands across the globe [3]. COVID-19 is a highly contagious disease that, as of January 2022, nearly 337 million confirmed cases have been reported, and over 5.5 million related deaths (5% global mortality rate) have been recorded globally [4,5,6]. The spread of COVID-19 appears to vary widely concerning the geographical area and patient characteristics. Data showed that the highest incidence rates have been in the U.S., South America, and Europe, while most African counties have reported comparatively lower numbers of confirmed COVID-19 cases than other parts of the world, which is hypothesized to stem from the high temperature and genetic characteristics of African people [7]. Males have been reported to be at higher risk of COVID-19 as well [8].

SARS-CoV-2 transmits through close contact by oral droplets. The infection begins when the virion binds the angiotensin-converting enzyme 2 (ACE2) receptor to interact with the spike protein (S protein) and TMPRSS2, a protease that cleaves the S protein to release its domains, which is an essential step in viral infection to enter the host cell [9]. Studies have revealed that severe disease forms tend to affect the elderly population and patients with comorbidities or poor immunity [10,11]. Moreover, patient characteristics are strong predictors of disease outcomes. Hypertension, diabetes mellitus, heart disease, renal disease, and cerebral stroke were commonly reported in patients with COVID-19 that did not survive [12]. Cytokine storm and mortality are higher in this type of population [7].

SARS-CoV-2 infection is thought to be mediated by activated T lymphocytes [13], which, in return, activate several inflammatory mediators, including interleukins, tumor necrosis factor-β (TNF-β), and chemokine (C-C motif) ligand 2 (CCL2) [14,15]. While the majority of patients are either asymptomatic or suffer from mild flu-like symptoms (such as shortness of breath, cough, and sore throat) [16], about 20% of cases show severe symptoms with pulmonary infiltrates that might develop into very serious complications [17]. COVID-19-associated severe infections are largely mediated by a cytokine storm, defined as a systemic inflammatory response with the excessive activation of immune cells and proinflammatory mediators such as FN-α, IL-1β, and IL-6 that lead to lung injury, then respiratory failure and death [15,16]. Moreover, inflammatory infiltration by macrophages and neutrophils is associated with an increased number of peripheral blood cells [18]. Patients with a cytokines storm can present with acute respiratory failure, sepsis, disseminated intravascular coagulation (DIC), and eventually death.

It is widely acknowledged that patients with respiratory viral illness are at a higher risk of secondary infections, whether bacterial or parasitic, which usually lead to a worse prognosis [19]. In the setting of COVID-19, the SARS-CoV-2 infection may be preceded by a prior microbial infection or have a concurrent or superinfection [20]. Previous reports have documented a significantly higher risk of microbial coinfection in SARS-CoV-2-positive patients [21]. The initial results from the United States (U.S.) and Europe found a significantly higher risk of mortality and severe illness among hospitalized patients with SARS-CoV-2 and bacterial coinfection [22,23]. However, later studies found contradictory results concerning the impact of coinfection on the outcomes of COVID-19; one study reported that the prevalence of bacterial infection was nearly 7%, and most secondary infections were associated with compromised patents [24]. Additionally, a recent meta-analysis found that the influenza viral coinfection did not significantly increase the risk of in-hospital mortality, while it significantly reduced the risk of critical illness [25]. A 2020 study reported that nearly one out of five COVID-19 cases are associated with primary or secondary infection, and an analysis supported a correlation between the presence of infection associated COVID-19 and poor prognosis [26]. 

Thus, we conducted the present literature review to provide updated evidence regarding the prevalence of coinfection and superinfection amongst patients with SARS-CoV-2, possible mechanisms underlying the higher risk of coinfection and superinfection in SARS-CoV-2 patients, and the impact of coinfection and superinfection on the outcomes of the patients with COVID-19.

## 2. Methodology

The present literature review was based on a comprehensive bibliographic search of Medline via the PubMed and Scopus databases through the period from December 2019 to January 2022. We used different combinations of the following keywords to retrieve the relevant records: COVID-19, severe acute respiratory syndrome coronavirus 2, SARS-CoV-2, secondary infection, coinfection, and superinfection. The search had no language or study design restrictions. We covered relevant publications assessing the prevalence of coinfection and superinfection amongst patients with SARS-CoV-2, possible mechanisms underlying the higher risk of coinfection and superinfection in SARS-CoV-2 patients, and the impact of coinfection and superinfection on the outcomes of the patients with COVID-19. Coinfection was defined as an infection that occurs concurrently with the SARS-CoV-2 infection. On the other hand, superinfection was defined as infections that follow the SARS-CoV-2 infection. We included studies with either community- or hospital-acquired coinfection/superinfection.

## 3. Pathogenesis COVID-19 Infection and the Role of Intrinsic Factors in Developing Severe Form of the Disease

Respiratory droplets are the primary means of human transmission of SARS-CoV-2, with an incubation period of one to two weeks [27]. The virulence mechanism of SARS-CoV-2 is widely believed to be mediated by binding to the ACE2 receptor, with subsequent ACE2 overexpression [28]. The increased ACE2 expression can lead to structural damage in the alveolar wall and hyperinflammatory status. It was noted that pulmonary edema and exudates are prominent in patients with COVID-19 [29]. Inflammatory factors were significantly elevated in a subpopulation of COVID-19 patients [30]; hyperinflammatory conditions of extreme sepsis have been well-mentioned [31]. Though, it is unclear to what level COVID-19-related inflammation is comparable or distinct from that usually seen in sepsis. Multiple case reports have indicated that patients with severe COVID-19 disorder display complications from hypercoagulability [32], as for microscopic thrombi and pulmonary emboli [33,34]. 

The main target of the SARS-CoV-2-induced cytokine storm is the lungs [35]. Tissue destruction due to the spread of SARS-CoV-2-infected cells can promote a dysfunctional immune response mediated mainly by macrophages that dysregulates the cytokine secretory pattern [36]. The virus’s invasion and replication are associated with a prompt release of proinflammatory cytokines and death of the cells, contributing to the release of the molecular patterns related to the damage and further amplifying the inflammatory response [37,38]. The exaggerated release of these cytokines is one of the ARDS mechanisms and multiple organ failure (MOF) in COVID-19 [39]. 

Tight intercellular communication usually occurs between lung epithelial cells expressing ACE2 and macrophages [40]. Macrophages have T-lymphocyte viral antigens, leading to T-cell subsets engagement and activation [41]. In response to the viral infection, an adaptive immune response with subsequent Th1 feature should release antiviral cytokines such as type I interferons (IFNs). Nevertheless, serious infections with coronavirus SARS may be associated with low IFN production levels, which have been previously documented [42]. 

The latest studies have shown elevated levels of inflammatory cytokines in patients with COVID-19, such as interleukins and tumor necrosis factor (TNF)-α [11] (Table 1). Moreover, fibroblast growth factor, granulocyte colony-stimulating factor (G-CSF), macrophage inflammatory protein 1, and granulocyte-macrophage colony-stimulating factor (GM-CSF) were reported to be elevated [43]. Interestingly, a significant correlation between the critical illness/intensive care unit (ICU) admission of COVID-19 patients and serum levels of IL-6 and TNF-α has been documented [44]. By interacting with its receptor, TNF-α may induce T-cell apoptosis, and IL-6 may suppress the activation of normal T cells that participate in the occurrence of lymphocytopenia. This feature is sometimes found in COVID-19 patients [45]. A recent study demonstrated a negative association between the total count of T cells, CD4+, and CD8+, and the TNF-α and IL-6 levels in ICU patients due to the COVID-19 [46]. 

In inflammatory sites, hypoxia is a common feature of multiple effects on the disease due to activating certain factors such as hypoxia-inducible factor-1α (HIF-1α) [47]. In COVID-19, HIF-1α overexpression can result in a cytokine storm by activating and stabilizing immune cells, with a subsequent release of inflammatory cytokines via vascular leakage and disruption of the alveolar–interstitial complex barrier [48,49].

Interestingly, there is increased evidence of the genetic role reporting the effect of common variants on the risk and COVID-19 severity (Table 1). Some of these variants affect the genes involved in innate immunity and inflammation through several pathways. This gene polymorphism has a different geographic distribution that could influence the impact of the infection, hence the different results that are reported by studies conducted in different countries [50,51].

The common variants of the two proteins most involved in the infection by SARS-CoV-2, namely ACE2 and Tmprss2, have different effects upon the infectiously and severity of COVID-19. Moreover, the factors that modulates these genes such as OM-85, a standardized lysate of bacteria, can be potential treatments [52,53,54] (Table 1). 

## 4. The Risk of Coinfections and Superinfections in COVID-19 Patients

It is imperative to quantify the risk of coinfection and superinfection, particularly bacterial infections, to properly address the role of prophylactic antibiotics in the management protocols of COVID-19 patients. The use of antimicrobial agents is now recognized as ineffective in the setting of COVID-19 and, thus, can lead to several misuse complications [62]. Nonetheless, if the risk of coinfection or superinfection is high, empirical antimicrobial agents can play a role, especially in patients with severe illness and an immunocompromised status [63]. However, the current practice of empirical antibiotics use in the COVID-19 setting seems to be based on the risk of coinfection with influenza virus rather than evidence-based quantification of the risk of coinfection or superinfection [64]. Thus, several reports have investigated the incidence of coinfection or superinfection in patients with SARS-CoV-2.

### 4.1. Prevalence of Coinfections

Concerning the rate of bacterial coinfections, a retrospective study retrieved the data of 16,654 deceased COVID-19 patients from Italy during the early peak of the pandemic (April 2020) and found that nearly 11% of the patients had bacterial superinfections [65]. This report was followed by myriad evidence from China that showed that bacterial coinfection incidences amongst adult COVID-19 patients were 0–16.7% [24]. For example, Liu et al. recruited 12 hospitalized patients with COVID-19 infection retrospectively and found that the rate of bacterial coinfections was 16.7% [66]. A larger retrospective study was conducted on 298 isolated COVID-19 cases in Shenzhen City and demonstrated that the rate of bacterial coinfections was 10.1% [67]. A notably higher prevalence of bacterial coinfection was reported in Thailand; out of 11 hospitalized patients, five patients (45.5%) had bacterial coinfections [68]. On the other hand, initial reports from the U.S found that the rate of bacterial coinfections ranged from 0 to 4.8% in critically ill patients with COVID-19 [69,70]. A comparatively higher rate of bacterial coinfections was reported among critically ill COVID-19 patients from Spain (12.5%) [71] (Table 2). 

However, the reports mentioned above were limited by small sample sizes and the lack of a clear definition of bacterial coinfections. Thus, subsequent large case series and retrospective studies have been conducted to reflect the prevalence of bacterial coinfections adequately. In a large cohort study of 5700 hospitalized patients from the U.S., the rate of bacterial coinfections was 0.3% [72]. A more recent report from Spain on 989 hospitalized patients noted a prevalence of 3.1% [73]. In a large cohort study from the U.S., swabs of 12,075 subjects who underwent SARS-CoV-2 testing were retrieved and tested for other pathogens. In SARS-CoV-2 patients, the rate of positive bacterial coinfection was high (55.4%) [74] (Table 2).

Additionally, a number of meta-analyses have tried to identify the exact prevalence of bacterial coinfections amongst patients with COVID-19. Initially, Langford et al. identified 24 retrospective studies with a total of 3506 patients; the rate of bacterial coinfection was 3.5% (95% confided interval (CI) 0.4–6.7%) [24]. However, this meta-analysis covered the studies published until the end of March 2020 only. Two subsequent meta-analyses found that the rate of bacterial coinfection ranged from 7% to 8% [75,76]. However, those meta-analyses were limited by small sample sizes of the included studies and the lack of a clear definition of coinfection. More recently, Musuuza et al. retrieved the data of 118 studies that covered both ICU and non-ICU patients. The pooled analysis revealed that the prevalence of bacterial coinfection was 8% (95% CI: 5–11%); the rate of coinfections was higher amongst non-ICU patients [26]. The observed heterogeneity in the published literature concerning the rate of bacterial coinfections may be attributed to the variations in the definition of coinfection, study design, and the study periods.

The published literature also shows notable heterogeneity in the distribution of causative agents of bacterial coinfections in the setting of SARS-CoV-2 infection. In Musuuza et al.’s meta-analysis, the following organisms accounted for nearly 30% of the cases: *Klebsiella pneumoniae*, *Streptococcus pneumoniae*, and *Staphylococcus aureus* [26]. A similar finding was reported by Contou et al. [77]. On the other hand, *Mycoplasma pneumonia* and *Pseudomonas aeruginosa* accounted for the majority of bacterial coinfections in other reports [75,76].

In terms of viral coinfections, Nowak et al. retrospectively collected the data of 1204 COVID-19 patients across New York City and reported that the rate of viral coinfections was 3%; the viral coinfections were mainly attributed to rhinovirus and influenza viruses [23]. Another Spanish report found that the rate of viral coinfection was 0.6%, mainly attributed to the influenza virus [73]. In Zhang et al. and Lv et al.’s reports, the viral isolations for the viral coinfections were mainly respiratory viruses and "atypical pathogens" [78,79]. To sum up, Musuuza et al.’s meta-analysis found that the rate of viral coinfections was 10% (95% CI: 6–14%), in which influenza virus and respiratory syncytial virus were the typical pathogens [26]. In a 2021 meta-analysis by Dadashi et al., a total of 11 studies (*n* = 3070 patients) were pooled to identify the prevalence of influenza virus coinfection in COVID-19 patients; the pooled analysis showed a prevalence of 0.8% [80].

For fungi, reports from China indicated that the rate of fungal coinfection amongst hospitalized patients ranged from 0 to 4% [81,82], commonly *Candida* species. In a meta-analysis study, Rawson et al. extracted the data of five studies reporting the rate of fungal coinfection in patients with confirmed SARS-CoV-2 during their hospital stay. The rate of fungal coinfection was found to be 8% [76]. In Musuuza et al.’s report, the rate of fungal coinfection was 8%, in which *Aspergillus* accounted for the majority of those coinfections [26] (Table 2).

Recently, a growing body of evidence tried to identify the rate of intestinal parasitic coinfection in SARS-CoV-2 patients. According to Wolday et al., the rate of parasitic coinfection in COVID-19 patients was 37.8% [83]. Another report from Egypt noted that parasitic coinfection presented in 72% and 20% of mild and critically ill cases, respectively [84]. On the contrary, other reports showed a very low rate of parasitic infections among COVID-19 patients [85].

Nonetheless, it should be noted that the abovementioned studies suffer from several methodological limitations considering the definition of coinfection, its differentiation from superinfection, and the methods of identification of the pathogens. Additionally, patients usually remain symptomatic for many days before hospitalization, making it more difficult to distinguish between coinfection and nosocomial infection. Moreover, the data collected in these studies are related to different countries with differences in socioeconomic statuses. In addition, the variations in the effect of the different SARS-CoV-2 variants were not considered (Table 2).

### 4.2. Prevalence of Superinfections

The initial reports during the early phase of the pandemic noted a considerable proportion of co-pathogens in COVID-19 patients, particularly in critically ill patients, which raised the possibility of a high risk of superinfection in COVID-19 patients [20]. Thus, several studies have tried to identify the exact risk of superinfections in COVID-19 patients. Reports from China showed that the rate of bacterial superinfection in SARS-CoV-2 patients can be as high as 42.2% [86]. Garcia-Vidal et al. investigated the rate of bacterial superinfection amongst 989 hospitalized patients in Spain and found that the rate was 4.7%; the causative agents were mainly *Pseudomonas aeruginosa* and *E. Coli* [73]. Another report from the U.K. found that the rate of bacterial superinfection was 9.3%, largely attributed to coagulase-negative *staphylococci* [22]. When these studies were combined with other reports, the pooled effect estimates showed a prevalence ranging from 14.3 to 24% [24,26]. For fungi, Hughes et al. retrospectively retrieved the data of 836 hospitalized patients with COVID-19 from two U.K. hospitals and found that fungal superinfection was common in this cohort (21.4%). Most fungal superinfections were attributed to *Candida* species [22]. However, these estimates should be interpreted cautiously, as most studies did not clearly distinguish between super- and coinfection or between community- and hospital-acquired infections. Additionally, the variations in the effects of the different SARS-CoV-2 variants were not considered.

## 5. Molecular Mechanism of Coinfection in COVID-19

The current literature lacks solid evidence considering the molecular mechanisms underlying the interactions between COVID-19 and coinfections. To date, the proposed mechanisms for microbial infections in patients with SARS-CoV-2 likely underpin superinfections rather than explaining the increased risk of coinfections.

One of the most widely accepted mechanisms for bacterial superinfection in SARS-CoV-2 relies on experimental investigations assessing the interactions between influenza virus and coinfections with other pathogens. The initial reports demonstrated significant synergistic interactions between influenza virus and *Streptococcus pneumoniae*, which results in a higher risk of bacterial pneumonia and considerable mortality and morbidity [87]. These interactions come predominantly from influenza virus-induced disruption of the respiratory epithelium and the overexpression of neuraminidase activity [88], leading to higher bacterial adherence coupled with the impaired clearance of *Streptococcus pneumoniae* due to the excessive expression of T-cell-mediated interferon-gamma [89]. These interactions favor bacterial adhesion and invasion into the respiratory epithelium and secondary bacterial pneumonia. Previous experimental evidence noted that nonlethal pneumococcal exposure can potentiate influenza infection and lead to death in mice [90]. Human studies have supported such evidence and reported a significant increase in the risk of death amongst patients with influenza infection and concurrent *Streptococcus pneumoniae* [87].

However, in the setting of SARS-CoV-2 infection, the mechanisms underlying virus-induced epithelial damage do not fully explain the increased risk of coinfection in patients with SARS-CoV-2. Previous authors hypothesized that SARS-CoV-2 plays a role in disturbing immunity and interferon counteraction towards bacterial colonization and proliferation; such a disruption is believed to stem from the hyperinflammatory status induced by SARS-CoV-2 infection and the overexpression of the NSP1 and ORF6 proteins. Besides, SARS-CoV-2-inducted airway dysfunction may further potentiate bacterial coinfection through the disruption of innate immunity [91]. The role of SARS-CoV-2 may also extend to genetic signaling within monocytes, which is attributed to similar actions of previous human coronaviruses. It was found that SARS-CoV dysregulates the expression of immune function-related genes in monocytes, particularly genes that stimulate interferon α/β and cathepsin/proteasome activities. Besides, it differentially regulates the genes responsible for Toll-like receptor (TLR) and other inflammatory mediator signaling, which establishes a suitable proinflammatory environment for bacterial coinfection [92]. Additional proposed mechanisms for bacterial coinfection in patients with SARS-CoV-2 include the distribution of the gut–lung axis by the virus and NL63-mediated enhanced adherence to virus-infected cell lines [93,94].

While the abovementioned mechanisms were postulated based on evidence regarding the interactions between bacterial coinfection and influenza or SARS-CoV viruses, SARS-CoV-2 appears to have distinct molecular mechanisms through its associated immunosuppression. As previously mentioned, the virulence mechanism of SARS-CoV-2 is mediated by binding to the ACE2 receptor expressed on the platelets, leading to a prothrombotic cascade of platelet activation, cellular aggregation, overexpression of the P-selectin and integrin, activation of GP11b/111a, and platelet spreading [92]. Other potential mechanisms are for platelet activation in patients with SARS-CoV-2 induction of platelet LR7 during the viremic phase [95]. Regardless of the underlying mechanism, platelet activation is likely to induce an immunosuppression status during the late stage of COVID-19 through overexpression of P-selectin glycoprotein ligand-1 (PSGL-1); microvascular occlusion through cellular aggregates; and excessive release of proinflammatory chemokines, cytokines, and IL-6 [20]. Besides, platelet-mediated activation of neutrophil extracellular traps (NETs) can exacerbate the risk of bacterial superinfection through their cytotoxic effects on the respiratory epithelium [96].

Lastly, the extensive use of antibiotics during the early waves of the pandemic might have particularly contributed to the development of the increased risk of bacterial superinfection in patients with SARS-CoV-2 and the development of resistant strains.

## 6. The Correlation of Coinfection with COVID-19 Severity and Outcomes

The assessment of the risk of coinfection and superinfection in SARS-CoV-2 patients should entail their impact on the outcomes of hospitalized patients, which would reflect the urge for investigating the presence of coinfection, monitoring superinfection, and even initiating empirical antimicrobial therapy. Bacterial coinfection and superinfection may negatively affect the outcomes of COVID-19 patients due to the expected lethal synergism previously observed with influenza virus [87]. Besides, bacterial superinfection may exaggerate the hyperinflammatory status, leading to a cytokine storm [91]. Two previous reports found that patients with bacterial coinfections were associated with in-hospital mortality. Likewise, amongst 289 hospitalized patients with COVID-19, the rate of bacterial coinfection was 8.7%, and they were found to have a higher risk of mortality, need for mechanical ventilation, or ICU admission. In Musuuza et al.’s meta-analysis, the risk of death was significantly higher amongst hospitalized COVID-19 patients than with patients without coinfection or superinfection (odds ratio (OR) = 3.31, *p* < 0.001). Notably, patients with superinfection had a higher incidence of mechanical ventilation than patients with coinfections, while patients with coinfections had longer hospital stays [26]. In a more recent review by Adalbert et al., the risk of mortality, need for mechanical ventilation, or ICU admission was notably high among COVID-19 patients with co-current *Staphylococcus aureus*.

In terms of viral coinfection, Guan et al. extracted the data from 12 studies that assessed the impact of influenza coinfection in patients with SARS-CoV-2. The pooled estimates found that the viral coinfection did not significantly increase the risk of in-hospital mortality, while it significantly reduced the risk of critical illness [25]. Likewise, Cheng et al. found no association between viral coinfection and in-hospital mortality [97]. In a recently reported real-world study from the U.S., patients with influenza coinfection were found to have a marginally higher risk of hospital admissions [98]. The trend towards a lower risk of severe COVID-19 in patients with influenza coinfection can be attributed to several factors. Firstly, influenza virus was found to stimulate the development of non-neutralizing antibodies that can bind to other pathogens, including SARS-CoV-2. This explanation is supported by evidence from previous reports that demonstrated a lower risk of severe COVID-19 in patients with a history of influenza vaccination; influenza vaccination may trigger a nonspecific immune response that can act to cover SARS-CoV-2. Another explanation is the observed reduction in the hyperinflammatory status amongst SARS-CoV-2 patients with influenza coinfection, contributing to a lower risk of severe outcomes [26].

Nonetheless, the favorable outcomes of COVID-19 in patients with influenza coinfection do not seem to extend to other viral infections. A recent review by Tsheten et al. concluded that patients with dengue coinfection had a high risk of mortality and critical illness. However, the included studies from this review were mostly case series, and further high-quality evidence is still needed to characterize the association between viral coinfection and SARS-CoV-2 outcomes [99].

While it is widely postulated that parasitic coinfection may reduce the severity of SARS-CoV-2 infection and has an inverse correlation with COVID-19-related adverse outcomes, only a few studies have investigated the impact of parasitic coinfection on the outcomes of COVID-19 patients. In a recent cohort study on 751 patients with SARS-CoV-2 from Ethiopia, patients with parasitic coinfection had a significantly lower risk of mortality than patients without the coinfection. Besides, COVID-19 patients with Malaria coinfection demonstrated good prognosis when compared with patients without malaria coinfection [100]. The authors postulated that this negative correlation is attributed to the lower risk of noncommunicable diseases in patients with parasitic coinfection and the effect of parasitic infection in levitating hyperinflammation [83].

## 7. Conclusions

Myriad evidence has investigated the risk and impact of coinfection and superinfection on patients with COVID-19 since the first wave of the pandemic but with conflicting results and ambiguous recommendations from the clinical guidelines. The currently published literature has shown a substantial variability concerning the rate of coinfection and superinfection in patients with SARS-CoV-2; nonetheless, their risk is considerable, and treatment protocols should consider the possibility of coinfection in hospitalized patients with SARS-CoV-2. Particularly, bacterial and viral coinfection/superinfection seem to have devastating consequences on the outcomes of COVID-19 patients and significantly increase the risk of mortality and critical illness. Thus, the presence of a coinfection should be meticulously investigated during the diagnosis of COVID-19 before initiating antimicrobial therapy. In the setting where the diagnosis of bacterial and viral coinfection/superinfection is not feasible, empirical antibiotic treatment can be considered. Still, it should be tailored according to the expected co-pathogens in the local setting, with strict adherence to antibiotic stewardship protocols. Additionally, many infections in the setting of SARS-CoV-2 appear to be nosocomial in nature, and hence, it is crucial to follow the infection control measures to reduce their incidence. Further large-sample, well-designed studies are warranted to investigate the prevalence and impact of COVID-19 coinfection, which can be followed by the recommendations of using empirical antimicrobial therapy in suspected patients based on these data.

While bacterial and viral coinfections tend to affect the outcomes of hospitalized COVID-19 patients negatively, the impact of a parasitic coinfection on the outcomes of COVID-19 patients is still unclear. Previous reports have demonstrated that parasitic coinfections may play a protective role against adverse outcomes in hospitalized COVID-19 patients. However, the evidence is still scarce, and further large studies should investigate the exact impact of parasitic coinfections on the outcomes of COVID-19 patients.

Finally, the emergence of different variants of SARS-CoV-2 and, consequently, their virulence should be taken into consideration when assessing and comparing studies from different socioeconomic backgrounds with different geographic locations. 

## Figures and Tables

**Table 1:** List of factors correlated with COVID-19 severity.

Factors	Level	Reference
Hypercoagulability	+	[55]
Cytokine storm	+	[56]
proinflammatory cytokines	+	[57]
interferons (IFNs).	−	[58]
tumor necrosis factor (TNF)-α	+	[59]
fibroblast growth factor, granulocyte colony-stimulating factor (G-CSF), macrophage inflammatory protein 1, and granulocyte-macrophage colony-stimulating factor (GM-CSF)	+	[59]
T-cell immunoglobulin and mucin-domain containing-3 (Tim-3) and programmed cell death protein-1 (PD-1)	+	[60]
Hypoxia-inducible factor-1α (HIF-1α)	+	[61]
Genes IL6 and AGT	+	[50]
Genetic intrinsic factors (MBL2, CD27, and TMPRSS2 variants)	+	[53]

+: increased, −: decreased.

**Table 2:** Prevalence of coinfection in the published literature.

Study	Study Design	Country	Setting	Number of Patients	Viral Coinfections (%)	Bacterial Coinfection *n* (%)	Fungal Coinfections (%)
Ding 2020	Case series	China	Non-ICU	115	4	0	0 (0)
Feng 2020	Case series	China	ICU and non-ICU	476	0		0 (0)
Garazzino 2020	Retrospective cohort	Italy	ICU and non-ICU	168	6		0 (0)
Gayam 2020	Case series	USA	ICU and non-ICU	350	0	0.3	0 (0)
Kim 2020	Retrospective cohort	USA	Non-ICU	115	22	0	0 (0)
Lian 2020	Retrospective cohort	China	ICU and non-ICU	788	NR	0	0 (0)
Lv 2020	Retrospective cohort	China	ICU and non-ICU	354	0.3	9	6 (2)
Mo 2020	Case series	China	ICU and non-ICU	155	8	1	0 (0)
Nowak 2020	Case series	USA	ICU and non-ICU	1204	3	0	0 (0)
Ozaras 2020	Case series	Turkey	ICU and non-ICU	1103	0.5	0	0 (0)
Richardson 2020	Case series	USA	ICU and non-ICU	5700	0.7	0.1	0 (0)
Wang L 2020	Case series	China	ICU and non-ICU	339	0	0.3	1 (0.3)
Wang R 2020	Case series	China	ICU and non-ICU	125	0.8		9 (7)
Wee 2020	Prospective cohort	Singapore	ICU and non-ICU	3807	0.08	NR	NR
Wu C 2020	Retrospective cohort	China	ICU and non-ICU	201	0.5		0 (0)
Yang X 2020	Case series	China	ICU	710	0	0.6	4 (0.6)
Zhang J 2020	Case series	China	ICU and non-ICU	140	1		1 (0.7)
Zhang G 2020	Case series	China	ICU and non-ICU	221	0.9		6 (3)
Zheng 2020	Case series	China	ICU and non-ICU	1001	0.2	NR	NR
Zhu 2020	Retrospective cohort	China	ICU and non-ICU	257	3		11 (4)
Chauhdary W 2020	Case series	Brunei Darussalam	ICU and non-ICU	141	NR	5	NR
Cheng L 2020	Retrospective cohort	Hong Kong	ICU and non-ICU	147	NR	3	NR
Cheng Y 2020	Retrospective cohort	China	ICU and non-ICU	213	46		NR
Cheng K 2020	Retrospective cohort	China	NR	212	NR	6	NR
Elabbadi A 2020	Case series	France	ICU	101	NR		NR
Falcone M 2020	Prospective cohort	Italy	ICU and non-ICU	315	NR		2 (1)
Garcia-Vidal 2021	Prospective cohort	Spain	ICU and non-ICU	989	1		7 (1)
Hazra A 2020	Retrospective cohort	USA	ICU and non-ICU	459	1	NR	NR
Hirotsu Y 2020	Prospective cohort	Japan	non-ICU	191	17	NR	NR
Hughes 2020	Case series	UK	ICU	836	NR	1	27 (3)
Kumar 2021	Retrospective cohort	USA	ICU and non-ICU	1573	NR		9 (1)
Lardaro T 2020	Retrospective cohort	USA	ICU and non-ICU	542	NR		NR
Lehmann C 2020	Retrospective cohort	USA	ICU and non-ICU	321	2	2	NR
Lendorf 2020	Retrospective cohort	Denmark	ICU and non-ICU	115	NR	8	1 (1)
Massey BW 2020	Case series	USA	ICU and non-ICU	12075	34.1–0.5	55.4-3.8	NR
Richardson S 2020	Case series	USA	ICU and non-ICU	5700	2.1	NR	NR
Li J 2020	Retrospective cohort	China	ICU and non-ICU	102	NR	56	NR

NR: Not reported.

## Data Availability

Not applicable.

## References

[1] Guo Y.R., Cao Q.D., Hong Z.S., Tan Y.Y., Chen S.D., Jin H.J., Tan K.S., Wang D.Y., Yan Y. (2020). The origin, transmission and clinical therapies on coronavirus disease 2019 (COVID-19) outbreak—An update on the status. Mil. Med. Res..

[2] Guan W.-J., Ni Z.-Y., Hu Y., Liang W.-H., Ou C.-Q., He J.-X., Liu L., Shan H., Lei C.-L., Hui D.S.C. (2020). Clinical Characteristics of Coronavirus Disease 2019 in China. N. Engl. J. Med..

[3] Al-Qahtani A.A. (2020). Severe Acute Respiratory Syndrome Coronavirus 2 (SARS-CoV-2): Emergence, history, basic and clinical aspects. Saudi J. Biol. Sci..

[4] World Health Organization (WHO) (2022). Coronavírus Disease 2019 (COVID-19): Situation Report-46.

[5] Romanov B.K. (2020). Coronavirus disease COVID-2019. Saf. Risk Pharmacother..

[6] World Health Organization (WHO) (2020). Coronavirus Disease 2019 (COVID-19): Situation Report-70 HIGHLIGHTS.

[7] Wamai R.G., Hirsch J.L., Van Damme W., Alnwick D., Bailey R.C., Hodgins S., Alam U., Anyona M. (2021). What Could Explain the Lower COVID-19 Burden in Africa despite Considerable Circulation of the SARS-CoV-2 Virus?. Int. J. Environ. Res. Public Health.

[8] Li Q., Guan X., Wu P., Wang X., Zhou L., Tong Y., Ren R., Leung K.S.M., Lau E.H.Y., Wong J.Y. (2020). Early Transmission Dynamics in Wuhan, China, of Novel Coronavirus–Infected Pneumonia. N. Engl. J. Med..

[9] Hoffmann M., Kleine-Weber H., Schroeder S., Krüger N., Herrler T., Erichsen S., Schiergens T.S., Herrler G., Wu N.H., Nitsche A. (2020). SARS-CoV-2 Cell Entry Depends on ACE2 and TMPRSS2 and Is Blocked by a Clinically Proven Protease Inhibitor. Cell.

[10] Wang W., Tang J., Wei F. (2020). Updated understanding of the outbreak of 2019 novel coronavirus (2019-nCoV) in Wuhan, China. J. Med. Virol..

[11] Huang C., Wang Y., Li X., Ren L., Zhao J., Hu Y., Zhang L., Fan G., Xu J., Gu X. (2020). Clinical features of patients infected with 2019 novel coronavirus in Wuhan, China. Lancet.

[12] Li X., Wang L., Yan S., Yang F., Xiang L., Zhu J., Shen B., Gong Z. (2020). Clinical characteristics of 25 death cases with COVID-19: A retrospective review of medical records in a single medical center, Wuhan, China. Int. J. Infect. Dis..

[13] Li G., Fan Y., Lai Y., Han T., Li Z., Zhou P., Pan P., Wang W., Hu D., Liu X. (2020). Coronavirus infections and immune responses. J. Med. Virol..

[14] Channappanavar R., Zhao J., Perlman S. (2014). T cell-mediated immune response to respiratory coronaviruses. Immunol. Res..

[15] Li X., Geng M., Peng Y., Meng L., Lu S. (2020). Molecular immune pathogenesis and diagnosis of COVID-19. J. Pharm. Anal..

[16] Prompetchara E., Ketloy C., Palaga T. (2020). Immune responses in COVID-19 and potential vaccines: Lessons learned from SARS and MERS epidemic. Asian Pac. J. Allergy Immunol..

[17] Mason R.J. (2020). Pathogenesis of COVID-19 from a cell biology perspective. Eur. Respir. J..

[18] Gu J., Gong E., Zhang B., Zheng J., Gao Z., Zhong Y., Zou W., Zhan J., Wang S., Xie Z. (2005). Multiple organ infection and the pathogenesis of SARS. J. Exp. Med..

[19] Arnold F.W., Fuqua J.L. (2020). Viral respiratory infections: A cause of community-acquired pneumonia or a predisposing factor?. Curr. Opin. Pulm. Med..

[20] Feldman C., Anderson R. (2021). The role of co-infections and secondary infections in patients with COVID-19. Pneumonia.

[21] Zhu X., Ge Y., Wu T., Zhao K., Chen Y., Wu B., Zhu F., Zhu B., Cui L. (2020). Co-infection with respiratory pathogens among COVID-2019 cases. Virus Res..

[22] Hughes S., Troise O., Donaldson H., Mughal N., Moore L.S.P. (2020). Bacterial and fungal coinfection among hospitalized patients with COVID-19: A retrospective cohort study in a UK secondary-care setting. Clin. Microbiol. Infect..

[23] Nowak M.D., Sordillo E.M., Gitman M.R., Paniz Mondolfi A.E. (2020). Coinfection in SARS-CoV-2 infected patients: Where are influenza virus and rhinovirus/enterovirus?. J. Med. Virol..

[24] Langford B.J., So M., Raybardhan S., Leung V., Westwood D., MacFadden D.R., Soucy J.P.R., Daneman N. (2020). Bacterial co-infection and secondary infection in patients with COVID-19: A living rapid review and meta-analysis. Clin. Microbiol. Infect..

[25] Guan Z., Chen C., Li Y., Yan D., Zhang X., Jiang D., Yang S., Li L. (2021). Impact of Coinfection With SARS-CoV-2 and Influenza on Disease Severity: A Systematic Review and Meta-Analysis. Front. Public Health.

[26] Musuuza J.S., Watson L., Parmasad V., Putman-Buehler N., Christensen L., Safdar N. (2021). Prevalence and outcomes of co-infection and superinfection with SARS-CoV-2 and other pathogens: A systematic review and meta-analysis. PLoS ONE.

[27] Bauch C.T., Lloyd-Smith J.O., Coffee M.P., Galvani A.P. (2005). Dynamically modeling SARS and other newly emerging respiratory illnesses: Past, present, and future. Epidemiology.

[28] Zhao Y., Zhao Z., Wang Y., Zhou Y., Ma Y., Zuo W. (2020). Single-cell RNA expression profiling of ACE2, the putative receptor of Wuhan 2019-nCov. bioRxiv.

[29] Tian S., Hu W., Niu L., Liu H., Xu H., Xiao S.-Y. (2020). Pulmonary Pathology of Early-Phase 2019 Novel Coronavirus (COVID-19) Pneumonia in Two Patients With Lung Cancer. J. Thorac. Oncol..

[30] Petrilli C.M., Jones S.A., Yang J., Rajagopalan H., O’Donnell L.F., Chernyak Y., Tobin K., Cerfolio R.J., Francois F., Horwitz L.I. (2020). Factors associated with hospitalization and critical illness among 4,103 patients with COVID-19 disease in New York City. medRxiv.

[31] Hotchkiss R.S., Karl I.E. (2003). The pathophysiology and treatment of sepsis. N. Engl. J. Med..

[32] Zhou F., Yu T., Du R., Fan G., Liu Y., Liu Z., Xiang J., Wang Y., Song B., Gu X. (2020). Clinical course and risk factors for mortality of adult inpatients with COVID-19 in Wuhan, China: A retrospective cohort study. Lancet.

[33] Danzi G.B., Loffi M., Galeazzi G., Gherbesi E. (2020). Acute pulmonary embolism and COVID-19 pneumonia: A random association?. Eur. Heart J..

[34] Tang N., Bai H., Chen X., Gong J., Li D., Sun Z. (2020). Anticoagulant treatment is associated with decreased mortality in severe coronavirus disease 2019 patients with coagulopathy. J. Thromb. Haemost..

[35] Pelaia C., Tinello C., Vatrella A., De Sarro G., Pelaia G. (2020). Lung under attack by COVID-19-induced cytokine storm: Pathogenic mechanisms and therapeutic implications. Ther. Adv. Respir. Dis..

[36] Tay M.Z., Poh C.M., Rénia L., MacAry P.A., Ng L.F.P. (2020). The trinity of COVID-19: Immunity, inflammation and intervention. Nat. Rev. Immunol..

[37] Siu K., Yuen K., Castano-Rodriguez C., Ye Z., Yeung M., Fung S., Yuan S., Chan C., Yuen K., Enjuanes L. (2019). Severe acute respiratory syndrome Coronavirus ORF3a protein activates the NLRP3 inflammasome by promoting TRAF3-dependent ubiquitination of ASC. FASEB J..

[38] Chen I.-Y., Moriyama M., Chang M.-F., Ichinohe T. (2019). Severe Acute Respiratory Syndrome Coronavirus Viroporin 3a Activates the NLRP3 Inflammasome. Front. Microbiol..

[39] Zhang W., Zhao Y., Zhang F., Wang Q., Li T., Liu Z., Wang J., Qin Y., Zhang X., Yan X. (2020). The use of anti-inflammatory drugs in the treatment of people with severe coronavirus disease 2019 (COVID-19): The Perspectives of clinical immunologists from China. Clin. Immunol..

[40] Qi F., Qian S., Zhang S., Zhang Z. (2020). Single cell RNA sequencing of 13 human tissues identify cell types and receptors of human coronaviruses. Biochem. Biophys. Res. Commun..

[41] Sarzi-Puttini P., Giorgi V., Sirotti S., Marotto D., Ardizzone S., Rizzardini G., Antinori S., Galli M. (2020). COVID-19, cytokines and immunosuppression: What can we learn from severe acute respiratory syndrome?. Clin. Exp. Rheumatol..

[42] Chen J., Subbarao K. (2007). The Immunobiology of SARS. Annu. Rev. Immunol..

[43] Rabaan A.A., Al-Ahmed S.H., Al Mutair A., Alhumaid S., Sule A.A., Tirupathi R., Fawzy M., Muhammad J., Khan A., Hasan A. (2021). Immunopathogenesis and immunobiology of SARS-CoV-2. Infez. Med..

[44] Okabayashi T., Kariwa H., Yokota S., Iki S., Indoh T., Yokosawa N., Takashima I., Tsutsumi H., Fujii N. (2006). Cytokine regulation in SARS coronavirus infection compared to other respiratory virus infections. J. Med. Virol..

[45] Gupta S., Bi R., Kim C., Chiplunkar S., Yel L., Gollapudi S. (2005). Role of NF-κB signaling pathway in increased tumor necrosis factor-α-induced apoptosis of lymphocytes in aged humans. Cell Death Differ..

[46] Diao B., Wang C., Tan Y., Chen X., Liu Y., Ning L., Chen L., Li M., Liu Y., Wang G. (2020). Reduction and Functional Exhaustion of T Cells in Patients With Coronavirus Disease 2019 (COVID-19). Front. Immunol..

[47] Jahani M., Dokaneheifard S., Mansouri K. (2020). Hypoxia: A key feature of COVID-19 launching activation of HIF-1 and cytokine storm. J. Inflamm..

[48] McClendon J., Jansing N.L., Redente E.F., Gandjeva A., Ito Y., Colgan S.P., Ahmad A., Riches D.W.H., Chapman H.A., Mason R.J. (2017). Hypoxia-Inducible Factor 1α Signaling Promotes Repair of the Alveolar Epithelium after Acute Lung Injury. Am. J. Pathol..

[49] Eckle T., Brodsky K., Bonney M., Packard T., Han J., Borchers C.H., Mariani T.J., Kominsky D.J., Mittelbronn M., Eltzschig H.K. (2013). HIF1A Reduces Acute Lung Injury by Optimizing Carbohydrate Metabolism in the Alveolar Epithelium. PLoS Biol..

[50] Feng S., Song F., Guo W., Tan J., Zhang X., Qiao F., Guo J., Zhang L., Jia X. (2022). Potential Genes Associated with COVID-19 and Comorbidity. Int. J. Med. Sci..

[51] Monticelli M., Mele B.H., Andreotti G., Cubellis M.V., Riccio G. (2021). Why does SARS-CoV-2 hit in different ways? Host genetic factors can influence the acquisition or the course of COVID-19. Eur. J. Med. Genet..

[52] Li J., Wang Y., Liu Y., Zhang Z., Zhai Y., Dai Y., Wu Z., Nie X., Du L. (2022). Polymorphisms and mutations of ACE2 and TMPRSS2 genes are associated with COVID-19: A systematic review. Eur. J. Med. Res..

[53] Monticelli M., Hay Mele B., Benetti E., Fallerini C., Baldassarri M., Furini S., Frullanti E., Mari F., Andreotti G., Cubellis M.V. (2021). Protective Role of a TMPRSS2 Variant on Severe COVID-19 Outcome in Young Males and Elderly Women. Genes.

[54] Pivniouk V., Pivniouk O., DeVries A., Uhrlaub J.L., Michael A., Pivniouk D., VanLinden S.R., Conway M.Y., Hahn S., Malone S.P. (2022). The OM-85 bacterial lysate inhibits SARS-CoV-2 infection of epithelial cells by downregulating SARS-CoV-2 receptor expression. J. Allergy Clin. Immunol..

[55] Abou-Ismail M.Y., Diamond A., Kapoor S., Arafah Y., Nayak L. (2020). The hypercoagulable state in COVID-19: Incidence, pathophysiology, and management. Thromb. Res..

[56] Tang Y., Liu J., Zhang D., Xu Z., Ji J., Wen C. (2020). Cytokine Storm in COVID-19: The Current Evidence and Treatment Strategies. Front. Immunol..

[57] Chen G., Wu D., Guo W., Cao Y., Huang D., Wang H., Wang T., Zhang X., Chen H., Yu H. (2020). Clinical and immunological features of severe and moderate coronavirus disease 2019. J. Clin. Investig..

[58] Huang K.J., Su I.J., Theron M., Wu Y.C., Lai S.K., Liu C.C., Lei H.Y. (2005). An interferon-gamma-related cytokine storm in SARS patients. J. Med. Virol..

[59] Merad M., Martin J.C. (2020). Author Correction: Pathological inflammation in patients with COVID-19: A key role for monocytes and macrophages. Nat. Rev. Immunol..

[60] Aghbash P.S., Eslami N., Shamekh A., Entezari-Maleki T., Baghi H.B. (2021). SARS-CoV-2 infection: The role of PD-1/PD-L1 and CTLA-4 axis. Life Sci..

[61] Tian M., Liu W., Li X., Zhao P., Shereen M.A., Zhu C., Huang S., Liu S., Yu X., Yue M. (2021). HIF-1α promotes SARS-CoV-2 infection and aggravates inflammatory responses to COVID-19. Signal Transduct. Target. Ther..

[62] Huttner B.D., Catho G., Pano-Pardo J.R., Pulcini C., Schouten J. (2020). COVID-19: Don’t neglect antimicrobial stewardship principles!. Clin. Microbiol. Infect..

[63] Alhazzani W., Møller M.H., Arabi Y.M., Loeb M., Gong M.N., Fan E., Oczkowski S., Levy M.M., Derde L., Dzierba A. (2020). Surviving Sepsis Campaign: Guidelines on the management of critically ill adults with Coronavirus Disease 2019 (COVID-19). Intensive Care Med..

[64] Klein E.Y., Monteforte B., Gupta A., Jiang W., May L., Hsieh Y.H., Dugas A. (2016). The frequency of influenza and bacterial coinfection: A systematic review and meta-analysis. Influenza Other Respir. Viruses.

[65] Istituto Superiore di Sanita (2020). Caratteristiche dei Pazienti Deceduti Positivi a COVID-19 in Italia. https://www.epicentro.iss.it/coronavirus/bollettino/Report-COVID-2019_9_aprile.pdf.

[66] Liu Y., Yang Y., Zhang C., Huang F., Wang F., Yuan J., Wang Z., Li J., Li J., Feng C. (2020). Clinical and biochemical indexes from 2019-nCoV infected patients linked to viral loads and lung injury. Sci. China. Life Sci..

[67] Cai Q., Huang D., Ou P., Yu H., Zhu Z., Xia Z., Su Y., Ma Z., Zhang Y., Li Z. (2020). COVID-19 in a designated infectious diseases hospital outside Hubei Province, China. Allergy.

[68] Pongpirul W.A., Mott J.A., Woodring J.V., Uyeki T.M., MacArthur J.R., Vachiraphan A., Suwanvattana P., Uttayamakul S., Chunsuttiwat S., Chotpitayasunondh T. (2020). Clinical Characteristics of Patients Hospitalized with Coronavirus Disease, Thailand. Emerg. Infect. Dis..

[69] Bhatraju P.K., Ghassemieh B.J., Nichols M., Kim R., Jerome K.R., Nalla A.K., Greninger A.L., Pipavath S., Wurfel M.M., Evans L. (2020). COVID-19 in Critically Ill Patients in the Seattle Region—Case Series. N. Engl. J. Med..

[70] Arentz M., Yim E., Klaff L., Lokhandwala S., Riedo F.X., Chong M., Lee M. (2020). Characteristics and Outcomes of 21 Critically Ill Patients With COVID-19 in Washington State. JAMA.

[71] Barrasa H., Rello J., Tejada S., Martín A., Balziskueta G., Vinuesa C., Fernández-Miret B., Villagra A., Vallejo A., San Sebastián A. (2020). SARS-CoV-2 in Spanish Intensive Care Units: Early experience with 15-day survival in Vitoria. Anaesth. Crit. Care Pain Med..

[72] Richardson S., Hirsch J.S., Narasimhan M., Crawford J.M., McGinn T., Davidson K.W., Barnaby D.P., Becker L.B., Chelico J.D., Cohen S.L. (2020). Presenting Characteristics, Comorbidities, and Outcomes Among 5700 Patients Hospitalized With COVID-19 in the New York City Area. JAMA.

[73] Garcia-Vidal C., Sanjuan G., Moreno-García E., Puerta-Alcalde P., Garcia-Pouton N., Chumbita M., Fernandez-Pittol M., Pitart C., Inciarte A., Bodro M. (2021). Incidence of co-infections and superinfections in hospitalized patients with COVID-19: A retrospective cohort study. Clin. Microbiol. Infect..

[74] Massey B.W., Jayathilake K., Meltzer H.Y. (2020). Respiratory Microbial Co-infection With SARS-CoV-2. Front. Microbiol..

[75] Lansbury L., Lim B., Baskaran V., Lim W.S. (2020). Co-infections in people with COVID-19: A systematic review and meta-analysis. J. Infect..

[76] Rawson T.M., Moore L.S.P., Zhu N., Ranganathan N., Skolimowska K., Gilchrist M., Satta G., Cooke G., Holmes A. (2020). Bacterial and Fungal Coinfection in Individuals With Coronavirus: A Rapid Review To Support COVID-19 Antimicrobial Prescribing. Clin. Infect. Dis. Off. Publ. Infect. Dis. Soc. Am..

[77] Contou D., Claudinon A., Pajot O., Micaëlo M., Longuet Flandre P., Dubert M., Cally R., Logre E., Fraissé M., Mentec H. (2020). Bacterial and viral co-infections in patients with severe SARS-CoV-2 pneumonia admitted to a French ICU. Ann. Intensive Care.

[78] Lv Z., Cheng S., Le J., Huang J., Feng L., Zhang B., Li Y. (2020). Clinical characteristics and co-infections of 354 hospitalized patients with COVID-19 in Wuhan, China: A retrospective cohort study. Microbes Infect..

[79] Zhang G., Hu C., Luo L., Fang F., Chen Y., Li J., Peng Z., Pan H. (2020). Clinical features and short-term outcomes of 221 patients with COVID-19 in Wuhan, China. J. Clin. Virol..

[80] Dadashi M., Khaleghnejad S., Abedi Elkhichi P., Goudarzi M., Goudarzi H., Taghavi A., Vaezjalali M., Hajikhani B. (2021). COVID-19 and Influenza Co-infection: A Systematic Review and Meta-Analysis. Front. Med..

[81] Chen N., Zhou M., Dong X., Qu J., Gong F., Han Y., Qiu Y., Wang J., Liu Y., Wei Y. (2020). Epidemiological and clinical characteristics of 99 cases of 2019 novel coronavirus pneumonia in Wuhan, China: A descriptive study. Lancet.

[82] Wu C., Chen X., Cai Y., Xia J., Zhou X., Xu S., Huang H., Zhang L., Zhou X., Du C. (2020). Risk Factors Associated With Acute Respiratory Distress Syndrome and Death in Patients With Coronavirus Disease 2019 Pneumonia in Wuhan, China. JAMA Intern. Med..

[83] Wolday D., Gebrecherkos T., Arefaine Z.G., Kiros Y.K., Gebreegzabher A., Tasew G., Abdulkader M., Abraha H.E., Desta A.A., Hailu A. (2021). Effect of co-infection with intestinal parasites on COVID-19 severity: A prospective observational cohort study. EClinicalMedicine.

[84] Abdel-Hamed E.F., Ibrahim M.N., Mostafa N.E., Moawad H.S.F., Elgammal N.E., Darwiesh E.M., El-rafey D.S., ElBadawy N.E., Al-Khoufi E.A., Hindawi S.I. (2021). Role of interferon gamma in SARS-CoV-2-positive patients with parasitic infections. Gut Pathog..

[85] Abd Al-Khaliq I., Mahdi I., Nasser A. (2021). Intestinal Parasitic Infections in Relation to COVID-19 in Baghdad City. Open Access Maced. J. Med. Sci..

[86] Wang L., He W., Yu X., Hu D., Bao M., Liu H., Zhou J., Jiang H. (2020). Coronavirus disease 2019 in elderly patients: Characteristics and prognostic factors based on 4-week follow-up. J. Infect..

[87] Klugman K.P., Chien Y.W., Madhi S.A. (2009). Pneumococcal pneumonia and influenza: A deadly combination. Vaccine.

[88] Peltola V.T., McCullers J.A., Fink R.J., Fedson D.S. (2004). Respiratory viruses predisposing to bacterial infections: Role of neuraminidase. Pediatr. Infect. Dis. J..

[89] Sun K., Metzger D.W. (2008). Inhibition of pulmonary antibacterial defense by interferon-gamma during recovery from influenza infection. Nat. Med..

[90] McCullers J.A., Rehg J.E. (2002). Lethal synergism between influenza virus and Streptococcus pneumoniae: Characterization of a mouse model and the role of platelet-activating factor receptor. J. Infect. Dis..

[91] Bengoechea J.A., Bamford C.G. (2020). SARS-CoV-2, bacterial co-infections, and AMR: The deadly trio in COVID-19?. EMBO Mol. Med..

[92] Manna S., Baindara P., Mandal S.M. (2020). Molecular pathogenesis of secondary bacterial infection associated to viral infections including SARS-CoV-2. J. Infect. Public Health.

[93] Golda A., Malek N., Dudek B., Zeglen S., Wojarski J., Ochman M., Kucewicz E., Zembala M., Potempa J., Pyrc K. (2011). Infection with human coronavirus NL63 enhances streptococcal adherence to epithelial cells. J. Gen. Virol..

[94] Zhang S., Liu Y., Wang X., Yang L., Li H., Wang Y., Liu M., Zhao X., Xie Y., Yang Y. (2020). SARS-CoV-2 binds platelet ACE2 to enhance thrombosis in COVID-19. J. Hematol. Oncol..

[95] Di Cristanziano V., Meyer-Schwickerath C., Eberhardt K.A., Rybniker J., Heger E., Knops E., Hallek M., Klein F., Holtick U., Jung N. (2021). Detection of SARS-CoV-2 viremia before onset of COVID-19 symptoms in an allo-transplanted patient with acute leukemia. Bone Marrow Transplant..

[96] Lv X., Wen T., Song J., Xie D., Wu L., Jiang X., Jiang P., Wen Z. (2017). Extracellular histones are clinically relevant mediators in the pathogenesis of acute respiratory distress syndrome. Respir. Res..

[97] Cheng Y., Ma J., Wang H., Wang X., Hu Z., Li H., Zhang H., Liu X. (2021). Co-infection of influenza A virus and SARS-CoV-2: A retrospective cohort study. J. Med. Virol..

[98] Chawla D., Chen X., Kuhlbusch K., Zalocusky K., Rizzo S. (2021). 281. Prevalence of Influenza Co-infection in a Real-world Cohort of COVID-19 Patients in the U.S. Open Forum Infect. Dis..

[99] Tsheten T., Clements A.C.A., Gray D.J., Adhikary R.K., Wangdi K. (2021). Clinical features and outcomes of COVID-19 and dengue co-infection: A systematic review. BMC Infect. Dis..

[100] Osei S.A., Biney R.P., Anning A.S., Nortey L.N., Ghartey-Kwansah G. (2022). Low incidence of COVID-19 case severity and mortality in Africa; Could malaria co-infection provide the missing link?. BMC Infect. Dis..

